# Iron Regulator Hepcidin Exhibits Antiviral Activity against Hepatitis C Virus

**DOI:** 10.1371/journal.pone.0046631

**Published:** 2012-10-22

**Authors:** Hongyan Liu, Thu Le Trinh, Huijia Dong, Robertson Keith, David Nelson, Chen Liu

**Affiliations:** 1 Department of Pathology, Immunology and Laboratory Medicine, University of Florida, Gainesville, Florida, United States of America; 2 Department of Biochemistry and Molecular Biology, Medical College of Georgia Cancer Center, Medical College of Georgia, Augusta, Georgia, United States of America; 3 Department of Medicine, University of Florida, Gainesville, Florida, United States of America; University of Tennessee Health Science Center, United States of America

## Abstract

Hepatitis C viral infection affects 170 million people worldwide. It causes serious chronic liver diseases. HCV infection has been implicated in iron accumulation in the liver and iron overload has been shown to be a potential cofactor for HCV associated hepatocellular carcinoma progression. The underlying mechanisms are not understood. Human hepcidin, a 25 amino acid peptide mainly produced by hepatocytes, is a key regulator of iron metabolism. Alteration of hepcidin expression levels has been reported in the setting of chronic HCV infection and hepatocellular carcinoma. In this study, we aim to examine the interactions between HCV infection and hepcidin expression in liver cells. We found that hepcidin expression was suppressed in HCV infected cells. The suppressive effect appears to be regulated by histone acetylation but not DNA methylation. Moreover, we found that hepcidin had a direct antiviral activity against HCV replication in cell culture. The antiviral effect is associated with STAT3 activation. In conclusion, hepcidin can induce intracellular antiviral state while HCV has a strategy to suppress hepcidin expression. This may be a novel mechanism by which HCV circumvents hepatic innate antiviral defense.

## Introduction

Hepatitis C virus (HCV) infects approximately 170 million people worldwide [1], [2]. It causes chronic liver diseases including chronic hepatitis, cirrhosis and hepatocellular carcinoma. HCV-associated chronic liver disease is the leading indication for liver transplantation [3]. Currently, there is no vaccine for HCV prevention; the standard therapy for HCV is pegylated interferon-alpha (IFN-α) plus ribavirin. However, the treatment results in sustained virologic response in approximately 50% patients with genotype 1 HCV infection, and is associated with significant side effect. Thus, understanding the pathogenesis of HCV and improvement of antiviral drugs for better efficacy for anti-HCV therapeutics are necessary.

Human hepcidin, also known as liver-expressed antimicrobial peptide-1, or LEAP-1, was first discovered and characterized as a highly disulfide-bonded peptide with antimicrobial activity [4]. Several studies have demonstrated that hepcidin has antimicrobial activity against bacteria and fungi [5], [6], [7]. Its major role appears to be in regulating iron homoeostasis. It is involved in the negative regulation of iron transport from intestinal enterocytes, reticuloendothelial macrophages, and hepatocytes to the plasma by binding it receptor ferroportin on the plasma membranes of these cells [8].

Iron is an essential element for all living organisms because it is required by a wide range of metabolic processes including DNA synthesis, oxygen transport, and energy production. However, excessive iron is harmful to the organism by evoking inflammatory cytokines, reactive oxygen species, liver fibrosis, and hepatic carcinogenesis [9], [10]. Chronic HCV infection can also result in iron accumulation in the liver, which possibly contributes to liver injury [11], [12]. However, little is known about the mechanism of iron accumulation in liver with HCV. A study has shown that HCV patients have low hepcidin levels in the liver [13], which raises the possibility that HCV inhibits hepcidin expression.

The control of hepcidin expression is complex. Oxidative stress can suppress hepcidin expression through inactivation of transcriptional factors including CCAAT/enhancer-binding protein α (C/EBPα) and signal transducer and activator of transcription 3 (STAT3) in alcohol-fed mice and in hypoxia treated cells [14], [15]. A recent study indicated that HCV-induced oxidative stress suppresses hepcidin expression in human hepatoma cell lines via histone deacetylase (HDAC) activation [16]. There are two signaling transduction pathways which are the major inducers of hepcidin expression: the hemojuvelin/bone morphogenetic protein/small mother against decapentaplegic homolog (HJV/BMP/SMAD) [17] and the STAT3 pathways [18], [19]. The STAT3 pathway is specifically involved in the inflammatory response linked with IL-6 [19]. STAT3 plays a role in innate immunity. We have previously shown that STAT3 activation is involved in efficient IFN induced anti-HCV activity in hepatocytes [20]. Other studies show that Daudi cells with defective STAT3 activation exhibit no IFN-induced anti-viral activities [21]. Considering the anti-bacterial activity of hepcidin and its expression during acute phase response, it is plausible that hepcidin plays a role in host innate immunity. Our study aims to elucidate the interaction of hepcidin and HCV infection.

## Materials and Methods

### Cell Lines and Patient Tissues

Huh7 cells were gifts from Dr. Christopher Seeger (Fox Chase Cancer Center, Philadelphia, PA) [22], Huh7.5 and FLneo cells were gifts from Dr. Charles M. Rice (Rockefeller University, New York, NY) [23], [24]. Huh7.5-JFH1 cells were prepared by electroporation of Huh7.5 with JFH1 RNA. pJFH-1 plasmid was a gift from Dr. Takaji Wakita (Department of Virology II, National Institute of Infectious Diseases, Tokyo, Japan) [25]. The linearized DNA was purified and used as a template for *in vitro* transcription using MEGAscript kit (Ambion, Austin, TX). All cell lines were grown in Dulbecco's modified Eagle medium, supplemented with 10% fetal bovine serum, 800 µM L-glutamine, 10 mM nonessential amino acids, and antibiotics at 37°C in 5% CO_2_. The primary hepatocytes were obtained from CellzDirect Inc. (Chapel Hill, NC). Human liver tissues were obtained at Shands Hospital according to an approved protocol for the institutional review board at the University of Florida. Written informed consents were obtained from all the subjects. Two patient samples were used for methylation assay, one was a 69 years old female in T2 stage of HCC, another one was a 55 years old male in T2 stage of HCC with HBV positive. For drug treatment, cell lines were treated with 300 nM Trichostatin A (TSA) for 24 h or with 5 µM 5-aza-2′-deoxycytidine (Aza) (Sigma, ST Louis, MO) for 3 days, changing Aza and medium every 24 h. Control cells were incubated with culture medium. To test the antiviral activity of hepcidin, the synthesized peptide (Bio-Synthesis, Lewisville, TX) or the scrambled peptide was added to the cultures at 20 µM. For virus infection, JFH1 virus (infectious genotype [GT]) 2a) or JC1 virus (GT2a/2a chimera) were added to the cell cultures and incubated for 16 h, and then cultured with new medium for another 3 days, or indicated time.

### RNA Extraction, Reverse Transcription-PCR (RT-PCR) and Quantitative Real-time PCR

RNA was extracted from cells or patient tissues using TRIzol RNA isolation reagent (Invitrogen, Carlsbad, CA). To prevent DNA contamination, total RNA was treated with RNase-free DNase II (Invitrogen, Carlsbad, CA). Human glyceraldehyde-3-phosphate dehydrogenase gene (*GAPDH*, forward primer 5′-TCACCAGGGCTGCTTTTA-3′ and reverse primer 5′-TTCACACCCATGACGAACA-3′) was used as an internal control in the PCR amplification. A two-step RT-PCR procedure was performed in all experiments. First, total RNA samples (1.6 µg per reaction) were reversely transcribed into cDNAs by RT II reverse transcriptase (Invitrogen). Then, the cDNAs were used as templates in PCR with hepcidin specific primers 5′-ACCAGAGCAAGCTCAAGACC-3′ and 5′-CAGGGCAGGTAGGTTCTACG-3′ or with HCV specific primers 5′-TTCACGCAGAAAGCGTCTAG-3′ and 5′-CACTCGCAAGCACCCTATCAGGCAG-3′. The primers for IFIT1 detection were 5′-TGGCTAAGCAAAACCCTGCA-3′ and 5′-TCTGGCCTTTCAGGTGTTTCAC-3′. The primers for OAS1 detection were 5′-AGGTGGTAAAGGGTGGCTCC-3′ and 5′-ACAACCAGGTCAGCGTCAGAT-3′. The amplification reactions were performed by using AmpliTaq Gold (Applied Biosystems, Foster City, CA), and PCR bands were visualized under UV light and photographed. Quantitative real-time PCR was also employed to measure the above genes for the indicated samples on step one plus real-time PCR system with SYBR Green MasterMix (Applied Biosystems).

### Bisulfite Genomic Sequencing

Genomic DNA was purified from cells and human liver specimens by the Wizard Genomic DNA Purification Kit (Promega, Madison, WI). 2 µg DNA was bisulfite modified by the EZ DNA Methylation Direct™ Kit (Zymo Research, Orange, CA). Sequence-specific primers to amplify the CpG rich regions in the hepcidin promoter were designed. The primers that were used for amplification were as follows: forward 5′-GGTGAGTAGTGTGTGTTTGTG-3′ and reverse 5′-CCAAACCACTAAACTCTCACC-3′. PCR products were amplified, purified and cloned into pGEM®-T Easy vector (Promega). Clones were selected through blue-white screening. Finally, the colonies harboring the insert were sequenced in a 96-well plate using the M13 reverse and/or forward primers.

### Cloning and Plasmid Construction

The full length hepcidin gene (NCBI accession number NM-021175) was cloned from a patient liver specimen. In brief, RNA was extracted using TRIzol RNA isolation reagent (Invitrogen). The cDNA was synthesized using RT II reverse transcriptase (Invitrogen), and 2 µl of cDNA was used for PCR amplification. Hepcidin gene was amplified with primers 5′-ACCAGAGCAAGCTCAAGACC-3′ and 5′-CAGGGCAGGTAGGTTCTACG-3′. The reaction was performed at 94°C for 3 min, followed by 38 cycles at 94°C for 40 s, 58°C for 30 s, 72°C for 45 s and finished with an extension at 72°C for 10 min. The PCR product was then purified and cloned into pEF6/V5-His-TOPO vector. The expression vector pTOPO-hepcidin and the anti-sense vector pTOPO-anti-hepcidin were sequenced using the BigDye Terminator V3.1 Kit from Applied Biosystems.

### Small Interfering RNA Transfection

To knockdown STAT3, synthesized small interfering RNA (siRNA) duplexes were purchased from Cell Signaling co. (Danvers, MA). To knockdown hepcidin, plasmid shRNA specific for hepcidin was purchased from Source Bioscience (Nottingham, UK). Cells were seeded in six-well plates 16 hours before transfection at a confluence of 50%, and transfected with 100 nM siRNA or 2 µg plasmid shRNA using Lipofectamine 2000 (Invitrogen) according to the manufacturer's instruction.

### Western Blot Analysis

Proteins were separated by SDS-PAGE (10% or 15% acrylamide), transferred to nitrocellulose membranes, and then blocked with 5% skim milk in a phosphate-buffered saline. Membranes were incubated with one of the following antibodies: mouse anti-hepcidin monoclonal antibody (1∶250; abcam), anti-STAT3 antibody (1∶250; Santa Cruz), anti-pSTAT3 antibody (1∶100; Santa Cruz), or anti-actin antibody (1∶8000; Sigma-Aldrich). After washing, membranes were incubated with peroxidase-conjugated goat anti-mouse immunoglobulin G or goat anti-rabbit immunoglobulin G (Sigma-Aldrich). Signals were detected by using the Supersignal® West Pico Chemiluminescent Substrate (PIERCE) according to the manufacturer's directions.

### Immunofluorescence

Cells were grown on glass cover slips and fixed with 5% acetic acid in ethanol. The cells were washed with phosphate-buffered saline and incubated with monoclonal antibody to HCV NS5A protein for 1 hour. The secondary antibody was FITC-labeled goat anti-mouse immunoglobulin G antibody. The nuclei were counterstained with 4′,6-diamidino-2-phenylindole (DAPI; Vector Laboratories Inc, Burlingame, CA), followed by examination under a fluorescence microscope (Olympus Imaging America Inc, Center Valley, PA).

## Results

### Hepcidin Expression is Suppressed by HCV

**Figure 1:**
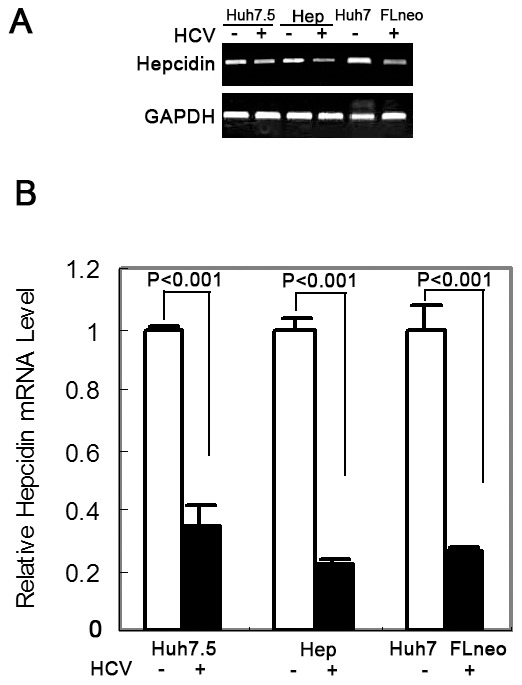
Hepcidin expression is decreased in HCV positive cells or HCV replicon cells. RT-PCR analysis (A) and qRT-PCR analysis (B) demonstrated hepcidin mRNA expression in control cells (Huh7.5, primary hepatocytes[Hep], and Huh7), JFH1 positive cells (Huh7.5 or primary hepatocytes) and HCV replicon cells (FLneo). The data are presented as mean ± SD from three independent experiments.

Previous studies have suggested the inhibitory effect of HCV infection on hepcidin expression [13], [16], [26]; however, one study has shown that hepcidin level is higher in patient serum with HCV than in controls [27]. To examine the link between hepcidin and HCV replication, we examined the effect of HCV replication on hepcidin expression using cell culture models: HCV infectious cell culture system (Huh7.5 JFH1-HCV), human primary hepatocytes infected with JFH1-HCV, and HCV replicon (FLneo). Hepcidin mRNA was determined using RT-PCR. The result showed that hepcidin mRNA expression was decreased significantly in all HCV positive cells compared to the three HCV negative cell lines (Fig. 1A). We also performed qRT-PCR and confirmed that HCV positive cells express less hepcidin compared to their respective controls (20% to 40% of control) (Fig. 1B).

### Hepcidin is Regulated by Histone Acetylation not DNA Methylation in HCV Positive Cells and in HCC

**Figure 2:**
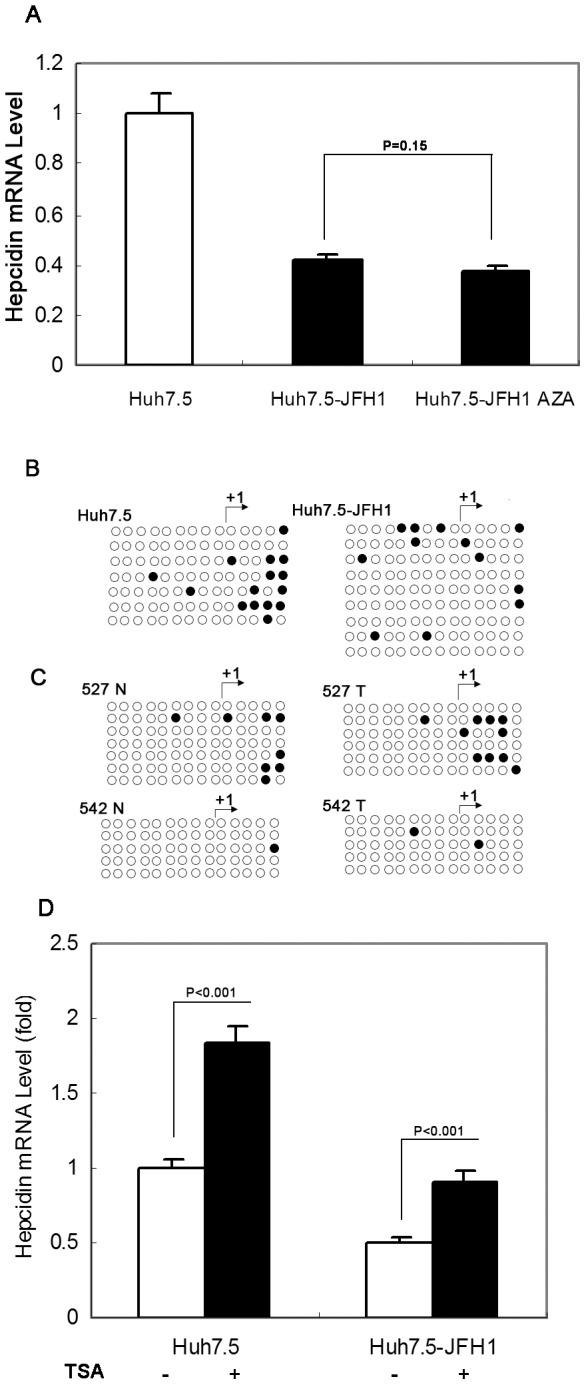
The regulation of hepcidin expression does not involve DNA methylation. (A) Huh7.5-JFH1 cells were treated with or without 5-aza-2′-deoxycytidine (Aza), and hepcidin expression was analyzed by qRT-PCR. *GAPDH* was used as the internal control in the PCR reaction. The data are presented as mean ± SD from three independent experiments; n.s., not significant. (B) and (C) Genomic DNA extracted from Huh7.5, Huh7.5-JFH1 cells (B) or two paired liver tissues (C) was modified with sodium bisulfate, PCR amplified, and subsequently cloned and sequenced. The methylation status at each CpG site in CpG island of hepcidin promoter is shown. Methylated sites are indicated by filled dark circles and unmethylated sites by empty white ones. N: nontumor tissues; T: tumor tissues. (D) Huh7.5-JFH1 cells were treated with or without Trichostatin A (TSA), and hepcidin expression was analyzed by qRT-PCR. The data are presented as mean ± SD from three independent experiments.

To investigate which mechanism regulates hepcidin expression in cells with HCV, we first examined whether DNA hypermethylation is involved in controlling hepcidin expression. We treated the JFH1 RNA transfected Huh7.5 cells with 5-azadeoxycytidine (Aza) and examined hepcidin expression by qRT-PCR. The result showed that there was no induction of hepcidin mRNA expression in the Huh7.5-JFH1 cell line after exposure to 5 µM of Aza for 3 days (Fig. 2A). We then examined the gene sequence of hepcidin. Although a 180 bp CpG rich region was identified in the hepcidin promoter, bisulfate genomic sequencing analysis of DNA methylation did not find differential methylation between the parental Huh7.5 cells and Huh7.5-JFH1 cells (Fig. 2B). Moreover, we investigated the methylation level in the representative sample sets from the HCC tissue which have lower expression levels of hepcidin in tumor tissues compared with the matched non-cancerous tissues (data not shown). None of the paired samples demonstrated differential methylation status in this CpG rich region (Fig. 2C). We further analyzed the effect of histone acetylation on hepcidin mRNA expression in Huh7.5 and Huh7.5-JFH1 cell lines using the nonspecific histone deacetylase inhibitor, Trichostatin A (TSA). TSA treatment for 24 h in Huh7.5 cells caused approximately a 1.84 fold elevation of hepcidin mRNA expression compared to untreated Huh7.5 cells and a 1.77 fold elevation of hepcidin mRNA expression in the Huh7.5-JFH1 cells when compared to its untreated control (Fig. 2D). Our finding suggests that hepcidin might be regulated by Histone acetylation. Other studies indicate that histone acetylation is involved in hepcidin down-regulation in HCV infected cells [16].

### Hepcidin Inhibits Subgenomic HCV RNA Replication

**Figure 3:**
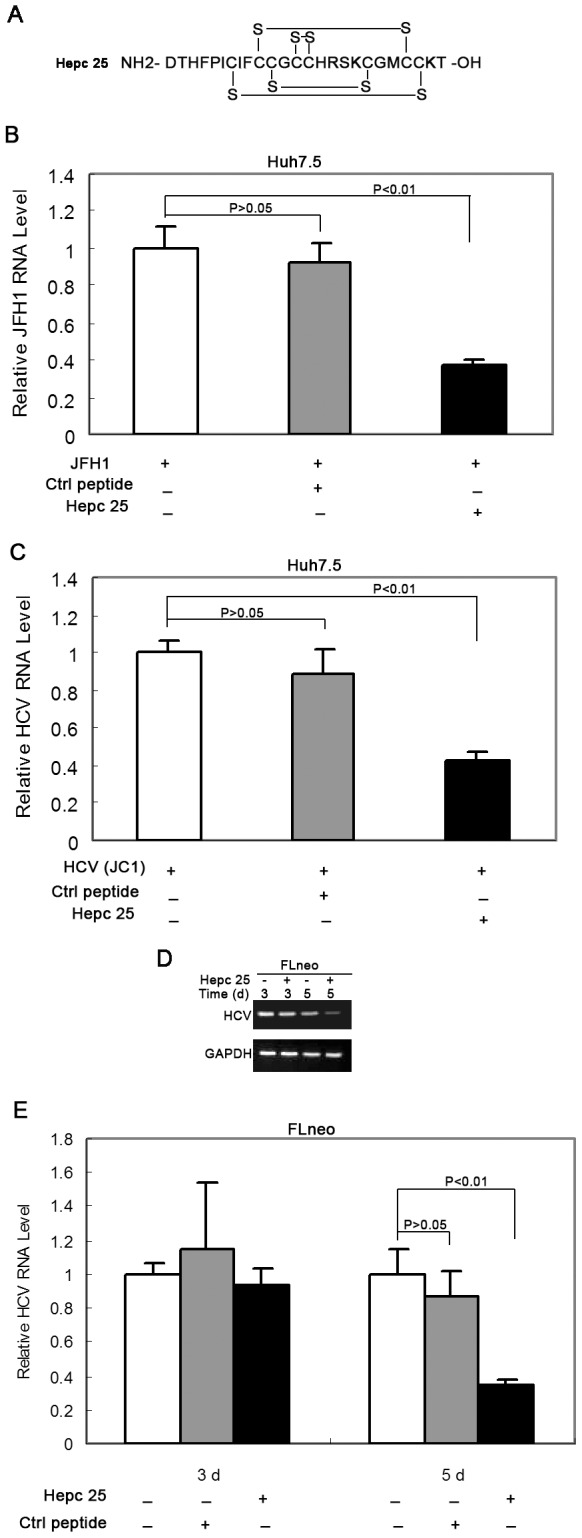
Hepcidin peptide inhibits HCV replication. (A) The synthesized hepcidin peptide with cyclized-disulfide bond between cysteine was shown. (B) and (C) Huh7.5 cells were incubated with JFH1 (B) or HCV-JC1 virus for 16 hours (C), then treated with or hepcidin peptide or control peptide for 3 days, and HCV mRNA levels were analyzed by qRT-PCR. *GAPDH* was used as the internal control in the PCR reaction. (D) and (E) FLneo cells were treated with or without hepcidin for 3 days and 5 days, and HCV mRNA expression was analyzed by RT-PCR (D) or qRT-PCR (E). The data are presented as mean ± SD from three independent experiments.

Our data clearly show that HCV inhibits hepcidin expression; we asked the question whether hepcidin has any impact on HCV replication. To investigate the effect of hepcidin on HCV-RNA expression in JFH1-infected Huh7.5 cells, we first incubated these cells with the synthesized hepcidin peptide (Fig. 3A). The cells were harvested for total RNA extraction after 3 days of treatment. QRT-PCR was performed to detect HCV RNA levels. The replication level of JFH1 HCV RNA was significantly attenuated by treatment with 20 µM of hepcidin peptide (Fig. 3B). To further confirm the inhibitory effect of hepcidin on HCV RNA replication, we also incubated JC1 virus infected Huh7.5 with hepcidin. Consistently, JC1 RNA expression was also reduced by treatment with hepcidin (Fig. 3C). To further confirm the anti-HCV activity of hepcidin, we then treated HCV genotype 1b replicon-containing cells, FLneo, with hepcidin for 3 days and 5 days. Total cellular RNA was extracted and subjected to RT-PCR and qRT-PCR for HCV RNA assay. As shown in figure 3D and 3E, hepcidin inhibited HCV RNA replication in full-length HCV replicon cells after 5 days of incubation. No effect of the scrambled peptide on HCV replication was observed after the cultured FL-Neo cells or the HCV infected Huh7.5 cells were treated with this control peptide. Collectively, these results indicate that hepcidin could trigger an antiviral effect in HCV cell culture systems.

**Figure 4:**
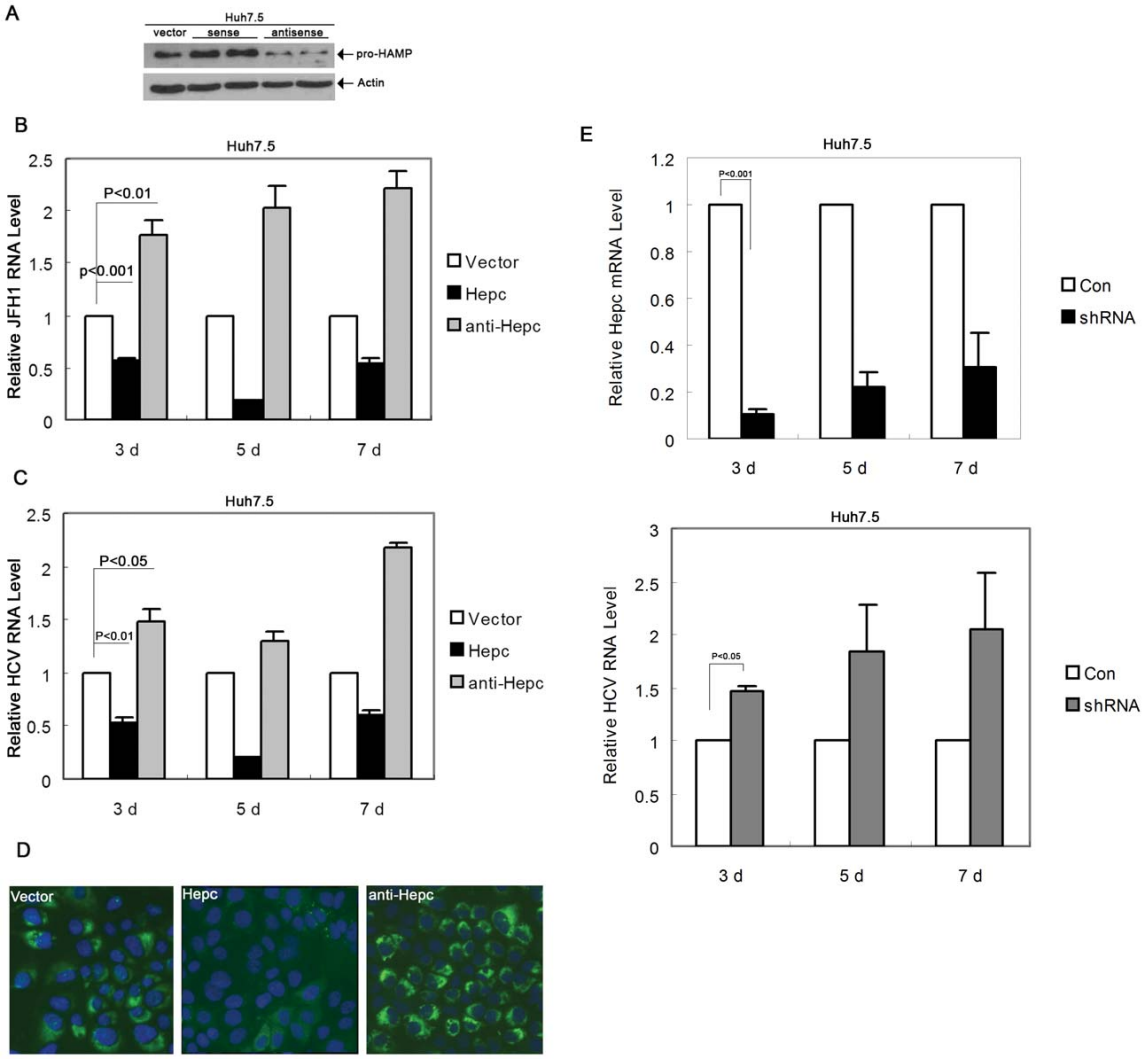
Modification of hepcidin expression in Huh7.5 cells affects HCV virus replication. (A) Western blot analysis of hepcidin expression in Huh7.5-vector, Huh7.5-Hepc and Huh7.5-antiHepc stable cell lines. (B,C) qRT-PCR analysis of HCV mRNA expression in JFH1 infected Huh7.5-vector, Huh7.5-hepc, Huh7.5-antihepc cells (B); and HCV-JC1 infected Huh7.5-vector, Huh7.5-hepc, and Huh7.5-antihepc cells (C). The data are presented as mean ± SD from three independent experiments. (D) Immunostaining for HCV (JC1) NS5A in Huh7.5-vector, Huh7.5-hepc and Huh7.5-antihepc cells on day 7 post infection. The original magnification is 200×. (E) Knockdown of hepcidin mRNA expression in Huh7.5 by shRNA transfection and endogenous hepcidin expression was analysed by qRT-PCR. The effect of hepcidin knockdown on HCV-JFH1 replication was assayed on day 3, day 5 and day 7 post-infection.

Since hepcidin is predominantly expressed in hepatocytes and HCV appears to inhibit its expression, we then asked the question whether exogenous over-expression of hepcidin would change the cell environment for HCV replication. To test this hypothesis, we established hepcidin expression stable cell line in Huh7.5 cells and then infected these cells with infectious HCV virus. Huh7.5 cells were stably transfected with the plasmid pTOPO-hepc, which expresses hepcidin protein. We also established another cell line that carries an antisense-silencing construct to suppress hepcidin expression. We performed immunoblot analysis to confirm over-expression or down-regulation of hepcidin protein in these cells (Fig. 4A). The stable cell lines were infected by JFH1 virus for 3, 5, and 7days, followed by total cellular RNA extraction and HCV RNA detection. When we introduced the sense plasmid to over-express hepcidin, JFH1 RNA levels decreased compared with those of control cells (cells transfected with empty vector) (Fig. 4B). In contrast, when we down-regulated the expression of hepcidin in cells, JFH1 RNA levels increased (Fig. 4B). To further investigate the effect of hepcidin on virus replication, we also incubated the stable cells with JC1 virus. Similarly, over-expression of hepcidin inhibited JC1 mRNA expression, while down-regulation of hepcidin enhanced JC1 mRNA expression (Fig. 4C). The results from immunostaining assay show that over-expression of hepcidin resulted in a significant decrease in JC1 protein level, whereas down-regulation of hepcidin led to an increase in JC1 protein expression (Fig. 4D). We also knocked down hepcidin transiently in Huh7.5 cells and then explored the effect of hepcidin down-modulation on HCV replication. Hepcidin shRNA could effectively knockdown hepcidin in Huh7.5 cells which showed a reduction of about 70% at the mRNA level at 72 hours post-transfection. Knockdown of hepcidin induced an increase of HCV replication in Huh7.5 cells (Fig. 4E).

### Hepcidin Antiviral Activity is Associated with STAT3 Activation

**Figure 5:**
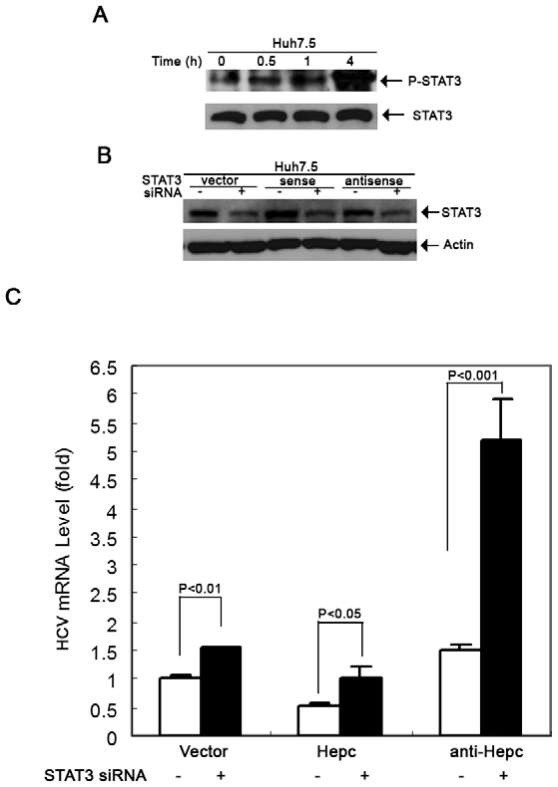
The antiviral activity of hepcidin is mediated by STAT3 activation. (A) Western blot analysis for pSTAT3 and total STAT3 in Huh7.5 control cells and Huh7.5 cells incubated with hepcidin for 0.5 h, 1 h and 4 h. (B) Western blot analysis for STAT3 in Huh7.5-vector, Huh7.5-hepc, Huh7.5-antihepc stable cell lines with or without STAT3 siRNA transfection. Actin was used as the loading control. (C) qRT-PCR analysis for HCV mRNA expression in the cells as described in (B). RNA was prepared from these cells after infected by JC1 virus for 3 days. The data are presented as mean ± SD from three independent experiments.

Although it is not completely understood how intracellular antiviral activity is established, JAK-STAT signaling has been known to play a key role in IFN-induced antiviral activity. We thus examined whether hepcidin induces antiviral activity through JAK-STAT signaling. Firstly, we tested whether hepcidin treatment can affect STAT1 and STAT3 phosphorylation. Huh7.5 cells were treated with 20 µM of hepcidin peptide for 30 min, 1 h and 4 h, followed by protein extraction. Then we analyzed STAT1 and STAT3 protein phosphorylation status by Western blot analysis using a monoclonal antibody specific for phosphorylated STAT or an antibody specific for total STAT. As shown in figure 5A, phosphorylated STAT3 was up-regulated by hepcidin treatment. Phosphorylated STAT1 was not detected (data not shown). The activation effect of hepcidin on STAT3 was time dependent and was more pronounced after 4 h incubation. To further determine whether the STAT3 pathway is relevant in hepcidin-induced antiviral activity in human hepatocytes, we specifically inhibited STAT3 using RNA interference technology in Huh7.5 cells and then treated these cells with JC1 virus. As shown in figure 5B, STAT3 siRNA could effectively knockdown STAT3 in Huh7.5-TOPO, Huh7.5-hepc and Huh7.5-antihepc cells. After knockdown of STAT3 in Huh7.5-TOPO cells the HCV-RNA levels were higher than those of control cells, suggesting that STAT3 has an inhibitory effect on viral replication (Fig. 5C). More importantly, STAT3 inhibition could reverse the inhibitory effect of hepcidin on HCV expression. Furthermore, when we down-regulated both STAT3 and hepcidin in Huh7.5 cells, we observed a 5-fold increase in HCV RNA expression which is higher than those observed in cells knockdown of a single gene only (Fig. 5C). Taken together, these findings suggest that the antiviral activity of hepcidin appears to be associated with the activation of the STAT3 pathway.

**Figure 6:**
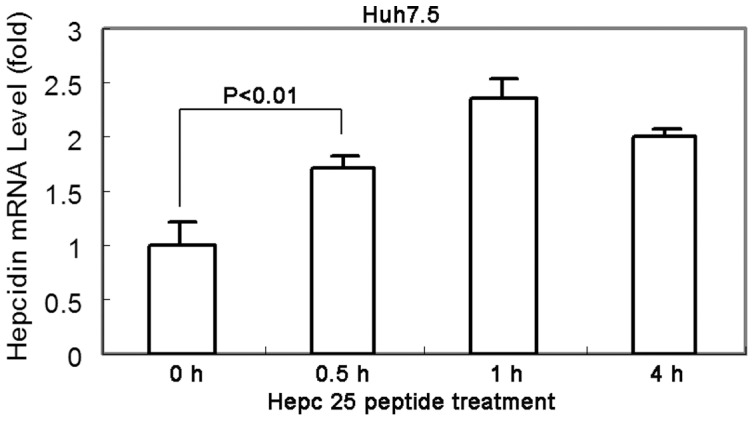
Hepcidin peptide treatment induces intracellular hepcidin expression. Huh7.5 cells were incubated with hepcidin for 0.5 h, 1 h and 4 h, followed by total RNA extraction. QRT-PCR was performed to examine hepcidin mRNA expression. The data are presented as mean ± SD from three independent experiments.

**Figure 7:**
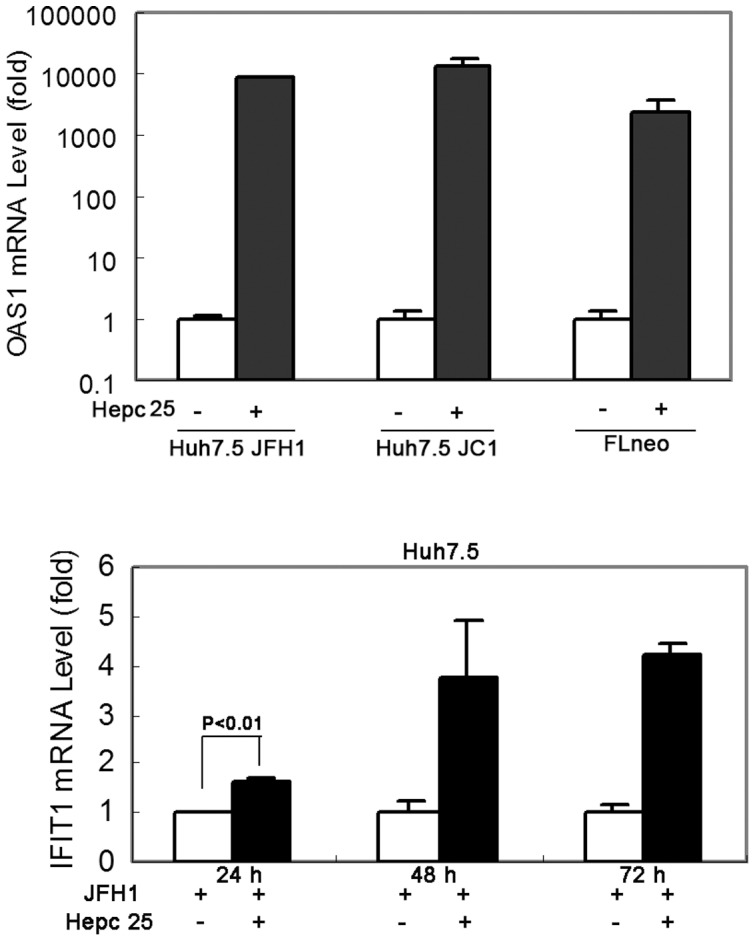
Hepcidin induces a classic interferon-induced gene 2′-5′-oligoadenylate synthetase 1 (OAS1) and interferon-induced protein with tetratricopeptide repeats 1 (IFIT1) expression. Huh7.5 cells were infected by HCV-JFH1 virus or JC1 virus, and treated with or without hepcidin peptide for 48 hours. FLneo was also treated with or without hepcidin peptide for 48 hours. OAS1 mRNA levels were analyzed by qRT-PCR in these cells. Huh7.5 cells were infected by HCV-JFH1 virus, and treated with or without hepcidin peptide for 24, 48 and 72 hours. IFIT1 mRNA levels were analyzed by qRT-PCR. *GAPDH* was used as the internal control in the PCR reaction.

STAT3 itself is needed for hepcidin expression [28] which regulates hepcidin expression through direct interaction with the STAT3 binding site localized in the proximal part of the hepcidin promoter. We observed that hepcidin peptide treatment induced endogenous hepcidin expression (Fig. 6). Expression of a classic IFN-induced gene, 2′,5′-oligoadenylate synthetase 1 (OAS1) was significantly increased after treatment with hepcidin. Further examination of downstream antiviral genes that are associated with IFN stimulation indicated that IFIT1 (ISG56) mRNA expression was induced by hepcidin (Fig. 7). However, there is no evidence of IFN induction by hepcidin (data not shown). These data show that the interferon inducible genes might be involved in the observed hepcidin mediated antiviral effect of STAT3.

## Discussion

As the key regulator of iron homeostasis, hepcidin binds to, internalizes, and degrades the iron exporter ferroportin [29], resulting in a decrease in serum iron concentration and an increase in intracellular iron content [30]. We and others [31] have demonstrated that the expression of hepcidin mRNA is suppressed in cancerous liver tissues from patients with HCC (data not published). Recently, studies have reported that alcohol consumption, a risk factor for HCC, can decrease hepcidin transcription and cause hepatic iron overload [14]. The experimental evidence in this study and other studies reveal that chronic viral hepatitis is also able to decrease hepcidin transcription (Fig. 1) [13], [16]. In addition, chronic hepatitis C virus infection results in excess iron accumulation in liver. The increased deposition of iron in the liver often triggers oxidative stress [32], inflammation and induces liver cell damage and cirrhosis [33]. Iron is an essential nutrient for cell growth and particularly required by cancer cells to proliferate [34]. Therefore, the down-regulation of hepcidin may stimulate tumor progression in chronic HCV infection patients.

We investigated how hepcidin is regulated in HCV infection. Our data suggests that DNA methylation does not appear to be the mechanism of hepcidin down-regulation in HCV infected cell lines or in the collected HCC patient samples. In contrast, histone acetylation may be a relevant epigenetic modulator of hepcidin in the process of HCV infection (Fig. 2), which is consistent with a previous report [16]. The molecular details on how this regulatory process occurs remains to be determined.

It has been reported that hepcidin has significant antimicrobial properties against Escherichia coli [5]. Hepcidin is a cysteine-rich molecular that contains four disulfide bonds and can also inhibit the growth of Salmonella typhimurium and Mycobacterium tuberculosis [35], [36]. Despite several studies on altered hepcidin expression in HCV infected human livers or serum [13], [27], [37], [38], [39], little is known about its antiviral effects. In this study, we found that hepcidin attenuated HCV expression in a hepatoma cell line containing HCV subgenomic replicon and reduced HCV viral replication in an infectious HCV cell culture model (Huh7.5). When we treated the JFH1 infected Huh7.5 cells with hepcidin peptide and examined JFH1 HCV RNA level, hepcidin significantly decreased JFH1 RNA expression (Fig. 3B). Similarly, hepcidin peptide inhibited another infectious HCV JC1 viral replication in Huh7.5 cells (Fig. 3C). It can also down-regulate HCV genotype 1b expression in FLneo HCV replicon cells (Fig. 3D and 3E). We further examined the anti-HCV activity of hepcidin by gene over-expression and knockdown. The data clearly show that over-expression of hepcidin reduced HCV expression, while knockdown of hepcidin induced viral RNA expression (Fig. 4). These results indicate that hepcidin might have a broad anti-HCV activity, at least for genotype 1 and genotype 2. The inhibitory effect appears to occur at the viral replication level, as replicon system does not have viral entry and viral packaging process. Taken together, these results suggest that both exogenous addition of hepcidin peptide and over-expression of hepcidin could attenuate HCV replication in cell models. Recently, there is a study suggesting that hepcidin is a cofactor for HCV replication [40] and studies also report that HAMP siRNA inhibits HCV replication [40], [41]. The different conclusions may result from the different cell culture models. They used Huh7 cells to test the effect of hepcidin silencing on JFH1 replication. Our results show that Huh7 cells express higher level of hepcidin than Huh7.5 cells (Fig. 1A). There is another possibility that the HAMP siRNA used in their study has off-target effects which affect HCV replication. Activation of the type I interferon pathway by siRNA is a major contributor to the off-target effects of RNA interference in mammalian cells. Various forms of siRNA have been reported to trigger IFN activation both *in vitro* and *in vivo* [42], [43], [44], [45], [46].

Besides its interaction with ferroportin, hepcidin also is known for its antimicrobial activity against bacteria and fungi [5], [6], [7]. It is a surprise to us that hepcidin exhibits direct anti-HCV effect in cell culture system. Our experiment did show that the antiviral effect is related to STAT3 activation (Fig. 5A). How hepcidin activates STAT3 remains to be determined. The possible mechanism is related to phosphorylation of JAKs. STAT3 knockdown experiment further confirmed its role in hepcidin-induced antiviral activity (Fig. 5C). The antiviral effect is similar to Interleukin-1 effect as we have previously reported [47]. We have to point out that how hepcidin activates STAT3 in the antiviral process in hepatocytes is unknown. Extensive experimentation is needed to determine the signaling events upon hepatocytes exposure to hepcidin. The other interesting aspect is the fact that STAT3 itself is needed for hepcidin expression [28]. It regulates hepcidin expression through direct interaction with the STAT3 binding site localized in the proximal part of the hepcidin promoter. Because hepcidin peptide treatment can induce cellular hepcidin expression (Fig. 6), it is possible that hepcidin has a positive feedback system to boost its antiviral effect.

We investigated the possibility that the antiviral activity of hepcidin is associated with intracellular antiviral state. The presence of IFN in the hepcidin treated cells was not directed, but some of the IFN-inducible genes, such as OAS1 and IFIT1 (ISG56), were significantly induced in hepcidin-treated cells (Fig. 7). It is possible that IFIT1 is directly involved in the hepcidin mediated antiviral effect. IFIT1 is known to be an important protein in intracellular antiviral state. Translation of the HCV positive-sense RNA genome is initiated by IRES-dependent ribosome recruitment, which requires eIF3 [48]. The direct binding of IFIT1 to eIF3 can inhibit HCV translation initiation both in vitro and within cells [49]. Future experiments should be performed to determine how hepcidin activates IFIT1 and what the mechanism of action is.

Our work demonstrates hepcidin effectively inhibits HCV replication in cell culture and HCV reduces hepcidin expression. It is plausible that hepcidin is a mediator in innate immunity and HCV has developed a strategy to suppress its expression. It is possible to develop a therapy using hepcidin. Besides its antiviral effect, the potential advantage of hepcidin therapy for HCV patients is restoration of iron homeostasis. It will be interesting to investigate the therapeutic efficacy of both antiviral activity and iron metabolism in small animal models or possible clinical studies. However, well-designed clinical studies addressing safety and long-term efficacy are needed in order to clarify the risks and benefits of hepcidin-targeted treatment.

In summary, we show for the first time that hepcidin can attenuate HCV replication and that this antiviral activity is through activation of STAT3. A strategy to augment hepcidin expression or hepcidin enhancing agents may be an effective therapy to treat patients with chronic hepatitis C, including those with existing interferon resistance.
